# Angiography-Derived Index of Microcirculatory Resistance Predicts Microvascular Obstruction and Infarct Size in Patients With STEMI

**DOI:** 10.1016/j.jacasi.2026.06.013

**Published:** 2026-08-01

**Authors:** Xincheng Sheng, Yumeng Hu, Yizhi Zhang, Jiyuan Chen, Jianping Xiang, Song Ding, Jun Pu

**Affiliations:** a Department of Cardiology, Punan Branch of Renji Hospital, Shanghai Jiaotong University School of Medicine, Shanghai, China; b Department of Cardiology, Shanghai Renji Hospital, Shanghai Jiaotong University School of Medicine, Shanghai, China; c Polytechnic Institute, Zhejiang University, Hangzhou, China; d ArteryFlow Research and Development Center for Intelligent Diagnosis and Treatment of Cardiovascular and Cerebrovascular Diseases, ArteryFlow Technology Co, Ltd, Hangzhou, China

**Keywords:** angiography-derived index of microcirculatory resistance, CMR-derived microvascular obstruction, infarct size, ST-segment elevation myocardial infarction

## Abstract

**Background:**

AccuIMR is a novel angiography-based method for assessing microvascular dysfunction (MVD) by simulating the index of microcirculatory resistance (IMR). In a prior study, AccuIMR showed a strong correlation with wire-based IMR.

**Objectives:**

This study aimed to evaluate the clinical significance of the angiography-derived index of microcirculatory resistance (AccuIMR) in patients with ST-segment elevation myocardial infarction (STEMI).

**Methods:**

This was a retrospective analysis of a prospective multicenter registry of STEMI patients who underwent cardiac magnetic resonance (CMR). This investigation included 482 STEMI patients. AccuIMR was obtained using angiographic images after percutaneous coronary intervention. The patients were categorized into the MVD group (AccuIMR >40) and the non-MVD group (AccuIMR ≤40). CMR results at 5 ± 2 days after percutaneous coronary intervention served as the reference standard for assessing CMR-derived microvascular obstruction (CMR-MVO) and infarct size.

**Results:**

Compared with the non-MVD group, the MVD group exhibited a larger CMR-MVO area, a larger infarct size area, and a lower left ventricular ejection fraction. Multivariate logistic regression analysis indicated that AccuIMR >40 was an independent predictive factor for CMR-MVO (OR: 4.730, 95% CI: 2.868-7.799, *P* < 0.001) and larger infarct size (OR: 2.101, 95% CI: 1.323-3.337, *P* = 0.002). DeLong's test, net reclassification index/integrated discrimination improvement, and likelihood ratio test demonstrated that incorporating AccuIMR into conventional predictive factors significantly improved the predictive accuracy for CMR-MVO and larger infarct size.

**Conclusions:**

AccuIMR is an efficient and dependable method for evaluating MVD in STEMI patients after percutaneous coronary intervention. When combined with conventional predictive factors, it provides supplementary information regarding cardiac function Registration: (AccuIMR Predicts Microvascular Obstruction and Infarct Size in STEMI Patients; NCT06947564).

Microvascular dysfunction (MVD) is a critical pathological change in coronary disease,^1^^,^^2^ which has a high incidence rate following percutaneous coronary intervention (PCI) in patients with ST-segment elevation myocardial infarction (STEMI).^3^ MVD may result in adverse clinical outcomes, including impaired cardiac function, an increased risk of heart failure, or subsequent cardiac mortality.^4^, ^5^, ^6^ Therefore, prompt diagnosis of MVD after PCI is of great significance, especially for patients with STEMI.

The index of microcirculatory resistance (IMR) is a reliable invasive approach achieved by using a pressure-temperature sensing guidewire.^7^ Wire-based IMR can evaluate the severity of MVD and identify STEMI patients at a higher risk,^8^, ^9^, ^10^ thereby supporting the adoption of more intensive therapeutic strategies during follow-up.^11^ However, the application of this method remains limited because of the risks of wire injury, the potential adverse effects of hyperemic agents, and the additional financial burden.

Recently, a novel angiography-based method for calculating IMR, angiography-derived index of microcirculatory resistance (AccuIMR), has been put forward.^12^ This method assesses MVD by simulating IMR using angiographic images, thus obviating the need for pressure-temperature sensing wires. In a prior study, AccuIMR showed a strong correlation with wire-based IMR values in STEMI patients, demonstrating high sensitivity and specificity.^13^ Nevertheless, given the limited sample size, it is too early to definitively determine the suitability of AccuIMR for STEMI patients. Additional research involving larger and more diverse samples is crucial to establish robust evidence for this application.

The objective of this study was to explore the clinical significance of AccuIMR in STEMI patients.

## Methods

### Study design

This was a retrospective, observational analysis aimed at evaluating the ability of AccuIMR to predict MVD and assess the degree of myocardial injury (NCT06947564). The study protocol was approved by the Ethics Committee of Renji Hospital, Shanghai Jiao Tong University School of Medicine.

The study data were collected from patients included in the EARLY-MYO-CMR (EARLY Assessment of MYOcardial; NCT03768453) registry between February 2014 and July 2020, which is a multicenter prospective registry that includes patients with STEMI who have undergone CMR examination.^14^, ^15^, ^16^, ^17^

### Participants

Patients were eligible for inclusion if they met the following criteria: 1) STEMI patients who had undergone PCI within 12 hours of symptom onset; 2) coronary microvascular function was evaluated using contrast-enhanced CMR within the first week after reperfusion; 3) no history of previous myocardial infarction or structural heart disease.

Thrombus aspiration, glycoprotein IIb/IIIa inhibitors (GPIs), and intracoronary vasodilators were administered at the investigator's discretion. All patients received dual antiplatelet therapy (DAPT) with aspirin 100 mg once daily plus clopidogrel 75 mg once daily or ticagrelor 90 mg twice daily.

STEMI was defined as patients presenting with chest pain and new ST-segment elevation, or new left bundle branch block on electrocardiography (ECG). The detailed description of the new ST-segment elevation indicated a new or presumed new ST-segment elevation of ≥1 mm in ≥2 anatomically contiguous leads (measured at the J-point) in all leads other than V_2_-V_3_ and ≥2 mm in men aged ≥40 years, ≥2.5 mm in men aged <40 years, and ≥1.5 mm in women regardless of age in leads V_2_-V_3_.^3^^,^^18^

### AccuIMR calculation

AccuIMR was calculated based on the angiographic images obtained immediately after PCI using a 6F guiding catheter. Based on the clinical significance of the IMR, we determined 40 as the optimal cutoff value for AccuIMR.^10^

The methodology for AccuIMR has been detailed in prior studies.^12^^,^^13^ Briefly, 2 angiographic projections separated by at least 25° and with optimal imaging quality were selected. A 3-dimensional reconstruction of the target vessel was then performed.

The TIMI frame count was analyzed to estimate the mean blood flow velocity by dividing the length of the reconstructed coronary tree by the average transport time.

AccuIMR was calculated in a blinded manner by 2 experienced investigators using dedicated software (AccuIMR, V1.0; ArteryFlow Technology). The formula for calculating AccuIMR is presented as follows:

AccuIMR = Pa × AccuFFRangio × L/V

where Pa is the mean aortic pressure; AccuFFRangio is the computed fractional flow reserve value; L is the length of the target vessel; and V is the mean flow velocity (Figure 1).

**Figure 1:**
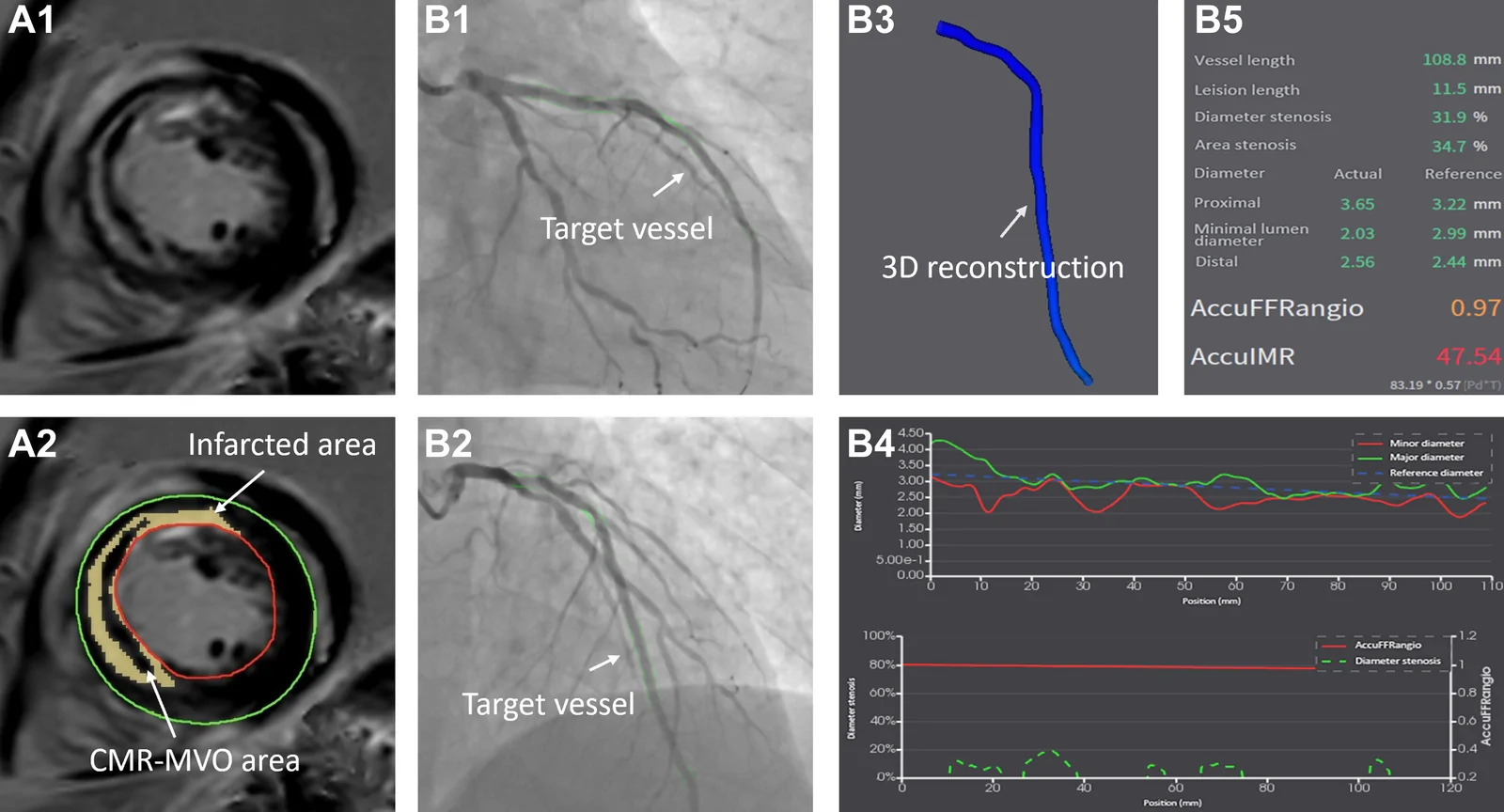
Contrast-Enhanced CMR and Calculation of AccuIMR (A_1_) The initial CMR late gadolinium enhancement image. (A_2_) The analyzed late gadolinium enhancement image. The green line represents the epicardial contour of the myocardium, the red line represents the endocardial contour of the myocardium, the yellow area represents the infarcted area, and the black area within the infarcted region represents the microvascular obstruction area. (B_1_ and B_2_) Two angiographic projections after PCI separated by at least 25°. (B_3_) Three-dimensional (3D) reconstruction of the target vessel. (B_4_) Luminal diameter and computed AccuIMR pullback. (B_5_) Computed AccuIMR. AccuIMR = angiography-derived index of microcirculatory resistance; CMR = cardiac magnetic resonance; CMR-MVO = cardiac magnetic resonance–derived microvascular obstruction.

### Cardiac magnetic resonance

Contrast-enhanced CMR was performed on a 3.0-T scanner (Achieva TX, Philips Healthcare) at 5 ± 2 days postreperfusion. All sequences were obtained during breath-hold, with a field of view of 350 × 350 mm^2^. Two experienced readers, who were blinded to the clinical data, evaluated the CMR results using validated software (QMass MR 7.5, Medis). The acquired images were used to assess ventricular parameters and calculate the left ventricular ejection fraction (LVEF).

Ten minutes after a bolus intravenous administration of the contrast agent (0.2 mmol/kg, Magnevist, Bayer HealthCare Pharmaceuticals), late gadolinium enhancement imaging was performed using a 3-dimensional (3D) inversion recovery segmented gradient echo sequence that covered the entire left ventricle (TR/TE: 3.5/1.7 ms, temporal resolution: 190 ms, voxel size: 1.5 × 1.7 × 10 mm^3^ interpolated into 0.74 × 0.74 × 5 mm^3^).^19^^,^^20^ Infarct size (IS) was identified as regions with hyperenhancement (signal intensity exceeding 5 standard deviations above normal myocardium) and quantified as a percentage of the left ventricular mass. CMR-derived microvascular obstruction (CMR-MVO) was defined as hypoenhanced areas within the infarcted region and further quantified as a percentage of the left ventricular mass.^17^^,^^19^ IS >25% was regarded as an indication of larger IS caused by STEMI, no 2^21^ (Figure 1).

CMR imaging was able to provide additional information for patients with STEMI. Previous studies have also verified the correlation between wire-based IMR and CMR-MVO in STEMI patient.^9^^,^^22^ Consequently, the results of CMR are considered a reliable benchmark for evaluating the clinical value of simulated IMR methods.

### Statistical analysis

Data are presented as mean ± SD when normally distributed or medians and IQRs when not normally distributed for continuous variables and as counts and percentages for categorical variables. Comparisons of continuous variables between 2 groups were conducted using independent-sample Student's *t*-tests, and categorical variables were analyzed using chi-square tests. The Spearman rank correlation coefficient was applied to evaluate the correlations between 2 continuous variables. Univariate logistic regression models were used to evaluate the variables associated with CMR-MVO and larger IS. Parameters that did not follow a normal distribution were categorized based on predefined cutoff values. Variables achieving a significance level of *P* < 0.1 were included in the multivariate logistic regression analysis. The area under the curve (AUC) obtained from receiver operating characteristic (ROC) analysis was utilized to assess the diagnostic accuracy. DeLong's test was employed to compare correlated AUCs derived from paired ROC analyses. To quantify the incremental value of AccuIMR beyond conventional risk factors, we calculated the net reclassification index (NRI) and integrated discrimination improvement (IDI) to assess statistical improvements in reclassification accuracy and discrimination. Decision curve analysis (DCA) was additionally performed to evaluate clinical utility by estimating net benefit across clinically relevant risk thresholds. Likelihood ratio test (LRT) and information criteria was also used to compare the nested models. A *P* value of <0.05 was considered statistically significant. All statistical analyses were conducted using SPSS Statistics (version 26, IBM Corp).

## Results

### Flow of participants

A total of 537 STEMI patients were initially enrolled in the study. Among them, 15 patients with suboptimal CMR images were subsequently excluded. After examining the angiographic images, 23 cases were excluded because of suboptimal coronary angiographic images, which included insufficient contrast opacification, unsatisfactory projection views, and severe overlap or distortion of the target vessel. Additionally, 17 cases were excluded because they did not have 2 angiographic projections separated by at least 25° apart post-PCI, which are necessary for AccuIMR computation. As a result, a total of 482 patients were enrolled in this study.

After calculating AccuIMR for all patients, we classified them into 2 groups according to the AccuIMR results. The MVD group consisted of 185 patients (38.4%) with AccuIMR >40, whereas the non-MVD group included 297 patients (61.6%) with AccuIMR ≤40 (Figure 2).

**Figure 2:**
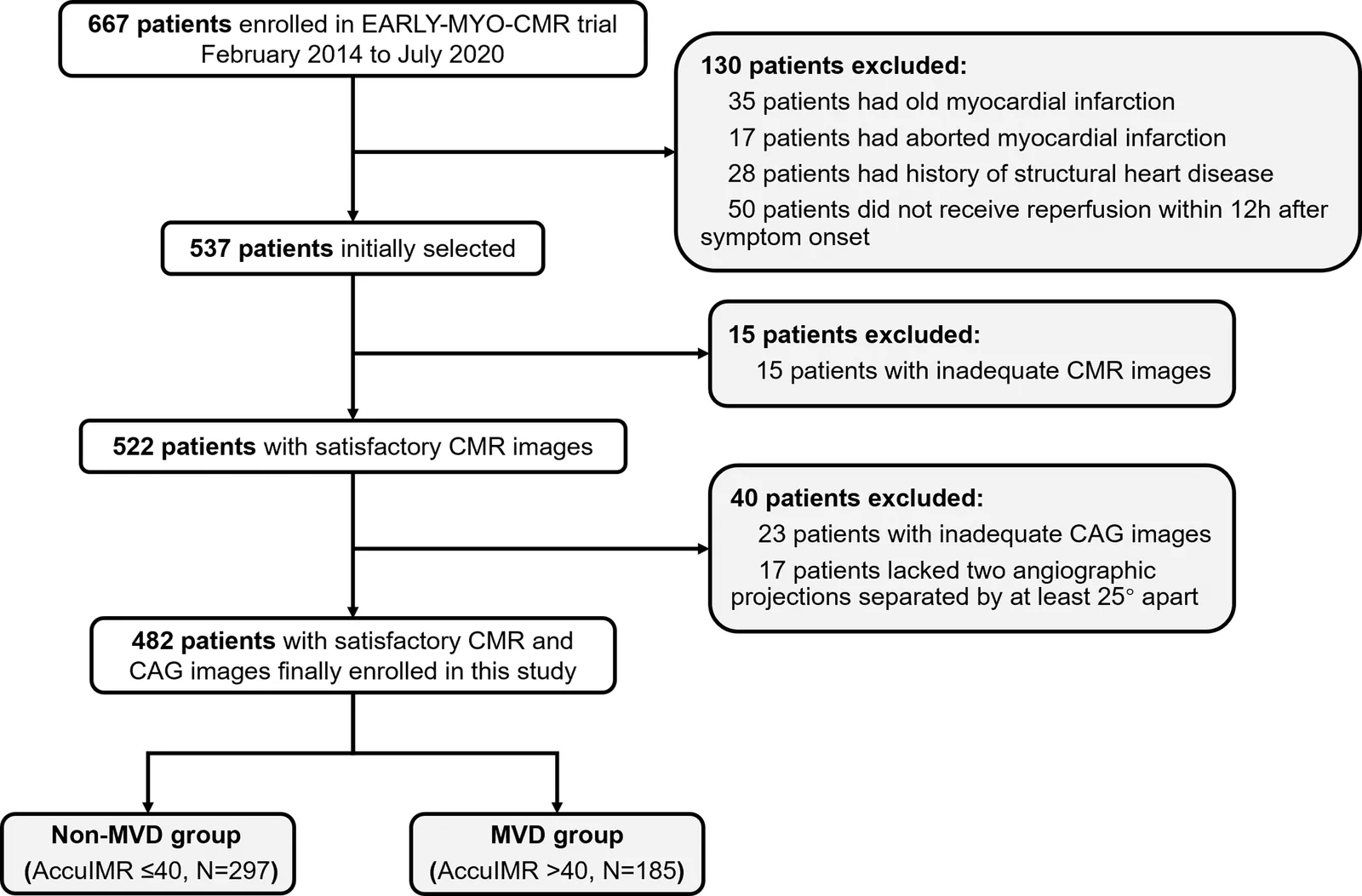
Study Flow Chart AccuIMR = angiography-derived index of microcirculatory resistance; CAG = coronary angiography; CMR = cardiac magnetic resonance; MVD = microvascular dysfunction.

### Baseline characteristics

The baseline and coronary angiography characteristics of patients in the 2 groups are presented in Table 1.

**Table 1:** Baseline and Coronary Angiography Characteristics of Patients in the Study Groups

	Non-MVD Group (AccuIMR ≤40, n = 297)	MVD Group (AccuIMR >40, n = 185)	*P* Value
Age, y	60.0 (55.0-65.5)	60.0 (53.0-65.0)	0.216
Male/female, n	264/33	165/20	1.000
BMI, kg/m^2^	24.7 (22.9-26.4)	24.6 (22.9-26.4)	0.753
Hypertension	169 (56.9%)	101 (54.6%)	0.658
Diabetes mellitus	90 (30.3%)	63 (34.1%)	0.462
Current smoking	200 (67.3%)	133 (71.9%)	0.293
White blood cells, ×10^9^/L	10.6 (8.89-13.1)	11.6 (9.56-13.5)	0.606
Hemoglobin, g/L	145 (136-156)	149 (137-157)	0.484
Platelets, ×10^9^/L	210 (174-246)	207 (172-244)	0.809
C-reactive protein, mg/L	6.81 (1.62-20.0)	5.75 (1.31-19.4)	0.966
ALT, U/L	32.0 (22.0-50.0)	41.0 (26.5-63.1)	0.188
Serum creatinine, μmol/L	73.7 (65.9-86.0)	74.0 (63.0-84.2)	0.461
Hemoglobin A1c, %	5.80 (5.50-6.60)	5.80 (5.40-7.00)	0.750
Peak troponin I, ng/mL	19.5 (4.09-43.0)	35.5 (6.19-70.8)	0.001
Peak CK-MB, ng/mL	181 (80.0-315)	295 (157-430)	<0.001
BNP, pg/mL	143 (60.0-297)	139 (54.2-286)	0.509
LDL-C, mmol/L	3.13 (2.52-3.72)	3.19 (2.61-3.74)	0.377
Pain-to-balloon time, h	4.50 (3.30-5.50)	4.80 (3.30-6.60)	0.077
Culprit vessel			0.031
LAD	164 (55.2%)	123 (66.5%)	
LCx	24 (8.1%)	15 (8.1%)	
RCA	109 (36.7%)	47 (25.4%)	
TIMI flow grade pre-PCI			0.921
0	219 (73.7%)	141 (76.2%)	
1	17 (5.7%)	10 (5.4%)	
2	23 (7.8%)	14 (7.6%)	
3	38 (12.8%)	20 (10.8%)	
TIMI thrombus grade			0.934
0	25 (8.4%)	15 (8.1%)	
1	5 (1.7%)	2 (1.1%)	
2	10 (3.4%)	4 (2.2%)	
3	9 (3.0%)	7 (3.8%)	
4	29 (9.8%)	16 (8.6%)	
5	219 (73.7%)	141 (76.2%)	
TIMI flow grade post-PCI			0.216
2	17 (5.7%)	16 (8.6%)	
3	280 (94.3%)	169 (91.4%)	
TMPG post-PCI			<0.001
0	1 (0.3%)	10 (5.4%)	
1	3 (1.0%)	5 (2.7%)	
2	46 (15.5%)	75 (40.5%)	
3	247 (83.2%)	95 (51.4%)	
Number of stents	1.19 ± 0.48	1.18 ± 0.50	0.976
Thrombus aspiration	74 (24.9%)	51 (27.6%)	0.518
GPI use	98 (33.0%)	69 (37.3%)	0.335
Intracoronary vasodilator	122 (41.1%)	89 (48.1%)	0.130
Nitroglycerin	107 (36.0%)	79 (42.7%)	0.143
Nicorandil	34 (11.4%)	30 (16.2%)	0.134
Fasudil	12 (4.0%)	12 (6.5%)	0.230
P2Y12 inhibitor			0.141
Clopidogrel	187 (63.0%)	104 (56.2%)	
Ticagrelor	110 (37.0%)	81 (43.8%)	

Data are presented as n (%) for categorical variables unless otherwise indicated and as mean ± SD when normally distributed or median (IQR) when not normally distributed for continuous variables.

AccuIMR = angiography-derived index of microcirculatory resistance; ALT = alanine aminotransferase; BMI = body mass index; BNP = B-type natriuretic peptide; CK-MB = creatine kinase–MB; GPI = glycoprotein IIb/IIIa inhibitor; LDL-C = low-density lipoprotein cholesterol; LAD = left anterior descending artery; LCx = left circumflex artery; MVD = microvascular dysfunction; PCI = percutaneous coronary intervention; RCA = right coronary artery; TMPG = TIMI myocardial perfusion grade.

There were no significant differences between the 2 groups in terms of sex, age, body mass index, chronic diseases like hypertension and diabetes mellitus, or pain-to-balloon time.

Additionally, multiple blood test indicators, including white blood cell count, hemoglobin, platelet count, C-reactive protein (CRP), alanine aminotransferase, serum creatinine, hemoglobin A1C, B-type natriuretic peptide, and low-density lipoprotein cholesterol (LDL-C), did not exhibit statistically significant differences between the 2 groups. The levels of peak troponin I (median [IQR]: 19.5 [4.09-43.0] vs 35.5 [6.19-70.8] ng/mL, *P* = 0.001) and peak creatine kinase–MB (CK-MB) (median [IQR]: 181 [80.0-315] vs 295 [157-430] ng/mL, *P* < 0.001) in the MVD group were significantly higher than those in the non-MVD group.

### Coronary angiography characteristics

In the MVD group, the left anterior descending artery (LAD) was more commonly identified as the culprit vessel. In contrast, in the non-MVD group, the right coronary artery (RCA) was more frequently the culprit vessel. The TIMI flow grade pre-PCI and TIMI thrombus grade were similarly distributed between groups. The number of stents deployed; utilization rates of thrombus aspiration, GPIs, and intracoronary vasodilators; as well as the selection of P2Y12 inhibitors (clopidogrel vs ticagrelor), were all comparable between groups. There was no significant disparity in the TIMI flow grade post-PCI between these 2 groups (94.3% [280/297] vs 91.4% [169/185], *P* = 0.216). Nevertheless, a significant difference in the TIMI myocardial perfusion grade (TMPG) post-PCI was noted between the groups (TMPG 3: 83.2% [247/297] vs 51.4% [95/185], *P* < 0.001).

### CMR results

The LVEF in the non-MVD group was significantly higher than that in the MVD group (median [IQR]: 56.3% [49.1%-62.9%] vs 50.0% [42.2%-56.6%], *P* < 0.001). CMR-MVO was detected in 286 patients. Among them, 137 patients were in the non-MVD group and 149 were in the MVD group. The CMR-MVO in the non-MVD group was significantly smaller than that in the MVD group (0.00% [0.00%-0.98%] vs 1.13% [0.24%-3.77%], *P* < 0.001). Similarly, the IS in the non-MVD group was significantly smaller than that in the MVD group (21.9% [14.0%-30.5%] vs 29.5% [22.0%-39.0%], *P* < 0.001) (Figure 3).

**Figure 3:**
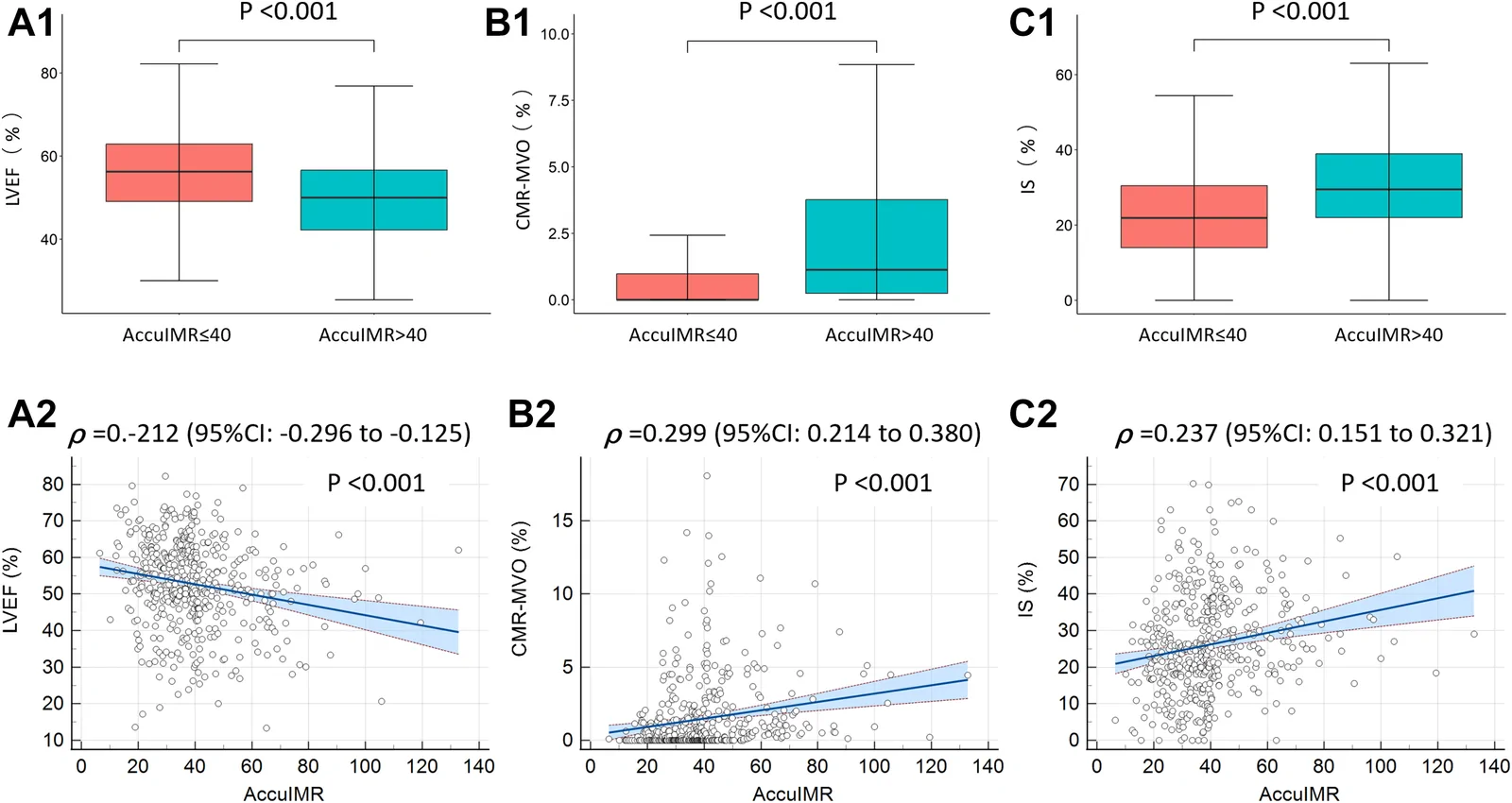
Association Between AccuIMR and Left Ventricular Ejection Fraction, CMR-MVO, and Infarct Size in STEMI Patients (A_1_) Left ventricular ejection fraction between the MVD group and the non-MVD group. (A_2_) Correlation between left ventricular ejection fraction and AccuIMR. (B_1_) CMR-MVO between the MVD group and the non-MVD group. (B_2_) Correlation between CMR-MVO and AccuIMR. (C_1_) Infarct size between the MVD group and the non-MVD group. (C_2_) Correlation between infarct size and AccuIMR. Bars in A_1_, B_1_, and C_1_ indicate IQR, the center line indicates median, and error bars indicate minimum and maximum values. AccuIMR = angiography-derived index of microcirculatory resistance; CMR-MVO = cardiac magnetic resonance–derived microvascular obstruction; IS = infarct size; LVEF = left ventricular ejection fraction.

In addition, the AccuIMR value exhibited an inverse correlation with LVEF (*ρ* = −0.212, 95% CI: −0.296 to −0.125, *P* < 0.001). On the contrary, AccuIMR was positively correlated with CMR-MVO (*ρ* = 0.299, 95% CI: 0.214-0.380, *P* < 0.001) and IS (*ρ* = 0.237, 95% CI: 0.151-0.321, *P* < 0.001) (Figure 3).

### Multivariate logistic regression analysis

Multivariate logistic regression analyses were performed to identify predictive factors of CMR-MVO and larger IS. Variables that were either conventional or shown to be relevant in previous studies were included in the analyses, including age, sex, body mass index, hypertension, diabetes mellitus, current smoking, serum creatinine, LDL-C, hemoglobin A1C, CRP, peak CK-MB, pain-to-balloon time, LAD as the culprit vessel, TIMI flow grade post-PCI, TMPG post-PCI, and AccuIMR value >40.

In the analysis of predictive factors for CMR-MVO, 7 variables, namely, age, sex, CRP, peak CK-MB, TIMI flow grade post-PCI, TMPG post-PCI, and AccuIMR >40 showed a *P* value of <0.1 in the univariate logistic analysis. Stepwise regression was employed to identify predictive factors for the multivariable model. As a result, sex (OR: 2.980, 95% CI: 1.440-6.168, *P* = 0.003), CRP (OR: 1.021, 95% CI: 1.009-1.033, *P* < 0.001), peak CK-MB (OR: 1.005, 95% CI: 1.003-1.006, *P* < 0.001), and AccuIMR >40 (OR: 4.730, 95% CI: 2.868-7.799, *P* < 0.001) were determined to be independent predictive factors for CMR-MVO (Table 2).

**Table 2:** Multivariable Logistic Analysis of the Predictive Factors for CMR-MVO

	Univariable Analysis		Multivariable Analysis	
	OR (95% CI)	*P* Value	OR (95% CI)	*P* Value
Age	0.970 (0.949-0.991)	0.005	—	—
Male	2.368 (1.319-4.252)	0.004	2.980 (1.440-6.168)	0.003
BMI	0.999 (0.935-1.068)	0.985	—	—
Hypertension	1.026 (0.705-1.494)	0.892	—	—
Diabetes mellitus	0.974 (0.653-1.452)	0.896	—	—
Current smoking	1.350 (0.856-2.131)	0.197	—	—
Serum creatinine	0.997 (0.988-1.006)	0.548	—	—
LDL-C	1.143 (0.941-1.390)	0.179	—	—
Hemoglobin A1c	1.061 (0.926-1.216)	0.396	—	—
C-reactive protein	1.020 (1.010-1.031)	<0.001	1.021 (1.009-1.033)	<0.001
Peak CK-MB	1.006 (1.004-1.037)	<0.001	1.005 (1.003-1.006)	<0.001
Pain-to-balloon time	1.022 (0.933-1.120)	0.639	—	—
Culprit vessel: LAD	0.924 (0.632-1.351)	0.683	—	—
TIMI flow grade post-PCI	0.418 (0.177-0.987)	0.047	—	—
TMPG post-PCI	0.441 (0.300-0.648)	<0.001	—	—
AccuIMR >40	5.298 (3.375-8.315)	<0.001	4.730 (2.868-7.799)	<0.001

AccuIMR = angiography-derived index of microcirculatory resistance; CMR-MVO = cardiac magnetic resonance–derived microvascular obstruction; BMI = body mass index; LDL-C = low-density lipoprotein cholesterol; CK-MB = creatine kinase–MB; LAD = left anterior descending artery; PCI = percutaneous coronary intervention; TMPG = TIMI myocardial perfusion grade.

In the univariate logistic analysis for assessing predictive factors for larger IS, several variables were identified with a *P* value < 0.1, including hemoglobin A1c, CRP, peak CK-MB, LAD as culprit vessel, TIMI flow grade post-PCI, TMPG post-PCI, and AccuIMR >40. After applying stepwise regression to identify the predictive factors for the multivariable model, CRP (OR: 1.012, 95% CI: 1.003-1.021, *P* = 0.012), peak CK-MB (OR: 1.002, 95% CI: 1.001-1.003, *P* < 0.001), LAD as culprit vessel (OR: 2.209, 95% CI: 1.429-3.414, *P* < 0.001), TMPG post-PCI (OR: 0.642, 95% CI: 0.435-0.950, *P* = 0.026), and AccuIMR >40 (OR: 2.101, 95% CI: 1.323-3.337, *P* = 0.002) were found to be independent predictive factors for larger IS (Table 3).

**Table 3:** Multivariable Logistic Analysis of the Predictive Factors for Larger Infarct Size

	Univariable Analysis		Multivariable Analysis	
	OR (95% CI)	*P* Value	OR (95% CI)	*P* Value
Age	1.014 (0.994-1.035)	0.160	—	—
Male	0.667 (0.373-1.194)	0.173	—	—
BMI	0.961 (0.901-1.025)	0.961	—	—
Hypertension	1.039 (0.722-1.495)	0.836	—	—
Diabetes mellitus	1.181 (0.801-1.741)	0.402	—	—
Current smoking	0.870 (0.562-1.344)	0.529	—	—
Serum creatinine	1.007 (0.998-1.016)	0.148	—	—
LDL-C	1.086 (0.901-1.310)	0.388	—	—
Hemoglobin A1c	1.144 (1.001-1.308)	0.049	—	—
C-reactive protein	1.015 (1.007-1.024)	<0.001	1.012 (1.003-1.021)	0.012
Peak CK-MB	1.003 (1.002-1.004)	<0.001	1.002 (1.001-1.003)	<0.001
Pain-to-balloon time	1.069 (0.980-1.166)	0.135	—	—
Culprit vessel: LAD	2.329 (1.596-3.397)	<0.001	2.209 (1.429-3.414)	<0.001
TIMI flow grade post-PCI	0.450 (0.213-0.951)	0.037	—	—
TMPG post-PCI	0.461 (0.328-0.647)	<0.001	0.642 (0.435-0.950)	0.026
AccuIMR >40	2.860 (1.948-4.197)	<0.001	2.101 (1.323-3.337)	0.002

AccuIMR = angiography-derived index of microcirculatory resistance; BMI = body mass index; LDL-C = low-density lipoprotein cholesterol; CK-MB = creatine kinase–MB; LAD = left anterior descending artery; PCI = percutaneous coronary intervention; TMPG = TIMI myocardial perfusion grade.

### Clinical usefulness of AccuIMR for predicting CMR-MVO and larger IS

We conducted further evaluations of the predictive value of AccuIMR for CMR-MVO and larger IS. Multivariable logistic regression models were developed by incorporating all conventional predictive factors. This included both prespecified covariates (age, sex, hypertension, diabetes mellitus, LDL-C level, and pain-to-balloon time) selected a priori based on clinical relevance, as well as additional variables that showed statistical significance in the initial multivariable logistic regression analysis.

When AccuIMR >40 was integrated into conventional predictive factors, the model incorporating AccuIMR exhibited significantly greater discriminatory power in predicting CMR-MVO (AUC: 0.803; 95% CI: 0.759-0.842 vs AUC: 0.766; 95% CI: 0.720-0.808; *P* = 0.025). The model with AccuIMR also demonstrated higher discriminatory power for predicting larger IS, yet no significance was detected (AUC: 0.788; 95% CI: 0.744-0.828 vs AUC: 0.773; 95% CI: 0.727-0.814; *P* = 0.104) (Figure 4).

**Figure 4:**
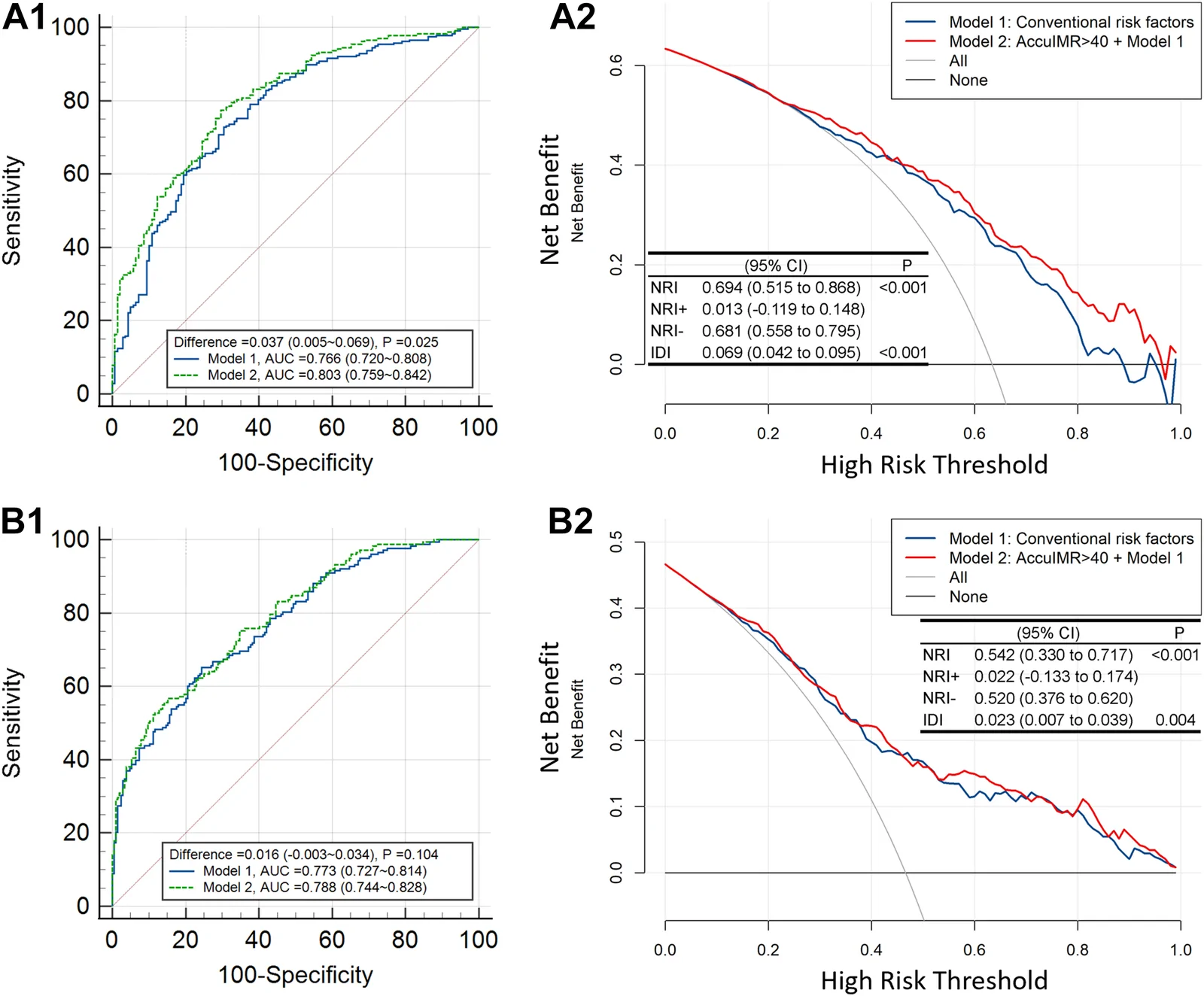
Incremental Predictive Value of AccuIMR Combined With Conventional Predictive Factors (A_1_) Comparison of the ROC curves between the models 1 and 2 for CMR-MVO prediction. (A_2_) The NRI and IDI analyses of AccuIMR for predicting CMR-MVO. Model 1 incorporates conventional predictive factors, including age, sex, hypertension, diabetes mellitus, LDL-C level, pain-to-balloon time, CRP, and peak CK-MB. Model 2 consists of conventional predictive factors plus AccuIMR >40. (B_1_) Comparison of the ROC curves between the 2 models for larger infarct size prediction. (B_2_) The NRI and IDI analyses of AccuIMR for predicting larger infarct size. Model 1 incorporates conventional predictive factors, including age, sex, hypertension, diabetes mellitus, low-density lipoprotein cholesterol, pain-to-balloon time, C-reactive protein, peak creatine kinase–MB, left anterior descending artery as culprit vessel, and TIMI myocardial perfusion grade post-PCI. Model 2 consists of the conventional predictive factors plus AccuIMR >40. AccuIMR = angiography-derived index of microcirculatory resistance. AUC = area under the curve; IDI = integrated discrimination improvement; NRI = net reclassification index; ROC = receiver operating characteristic.

The NRI and IDI analyses indicated a significant benefit in reclassification ability and incremental discrimination ability when AccuIMR >40 was incorporated into the conventional predictive factors model for both CMR-MVO (NRI: 0.694; 95% CI: 0.515-0.868; *P* < 0.001; IDI: 0.069; 95% CI: 0.042-0.095; *P* < 0.001) and larger IS (NRI: 0.542; 95% CI: 0.330-0.717; *P* < 0.001; IDI: 0.023; 95% CI: 0.007-0.039; *P* = 0.004). The larger area-under-the-decision curve for model 2 indicated greater clinical utility after incorporation of AccuIMR >40 (Figure 4).

To further evaluate the incremental predictive value of AccuIMR, we compared the nested models using the LRT and information criteria. When evaluating the predictive performance for CMR-MVO, the addition of AccuIMR >40 to the baseline clinical model demonstrated an overwhelming improvement in model fit. The likelihood ratio test revealed a highly significant enhancement (LRT chi-square = 32.30, *P* < 0.001). Most notably, the inclusion of AccuIMR led to dramatic reductions in both information criteria, with the Akaike information criterion (AIC) dropping from 434.87 to 404.57 (ΔAIC = −30.30) and the Bayesian information criterion (BIC) dropping from 466.32 to 439.96 (ΔBIC = −26.37). This profound improvement directly aligns with the pathophysiological mechanism, as IMR specifically quantifies microcirculatory resistance, making it an exceptionally robust and independent determinant for predicting CMR-MVO.

When evaluating the predictive performance for larger IS, the addition of AccuIMR to the baseline clinical model resulted in a statistically significant improvement in model fit (LRT chi-square = 11.24, *P* < 0.001). Furthermore, the inclusion of AccuIMR optimized the model's information profile. The AIC decreased substantially from 445.36 to 436.11 (ΔAIC = −9.24), and the BIC decreased from 484.81 to 479.51 (ΔBIC = −5.30). These consistent decreases in both AIC and BIC, which penalize for model complexity, strongly demonstrate that AccuIMR provides robust, independent additive value for predicting larger IS.

## Discussion

This cohort study demonstrates the diagnostic accuracy and clinical significance of AccuIMR in STEMI patients after PCI, with CMR serving as the reference standard (Central Illustration). The major findings of this study are as follows: 1) The AccuIMR measurement can be relatively easy to perform in STEMI patients after PCI. 2) AccuIMR is a reliable approach for evaluating MVD in STEMI patients after PCI. 3) In addition to other conventional predictive factors, AccuIMR can provide supplementary information regarding cardiac function in STEMI patients.

**Central Illustration:**
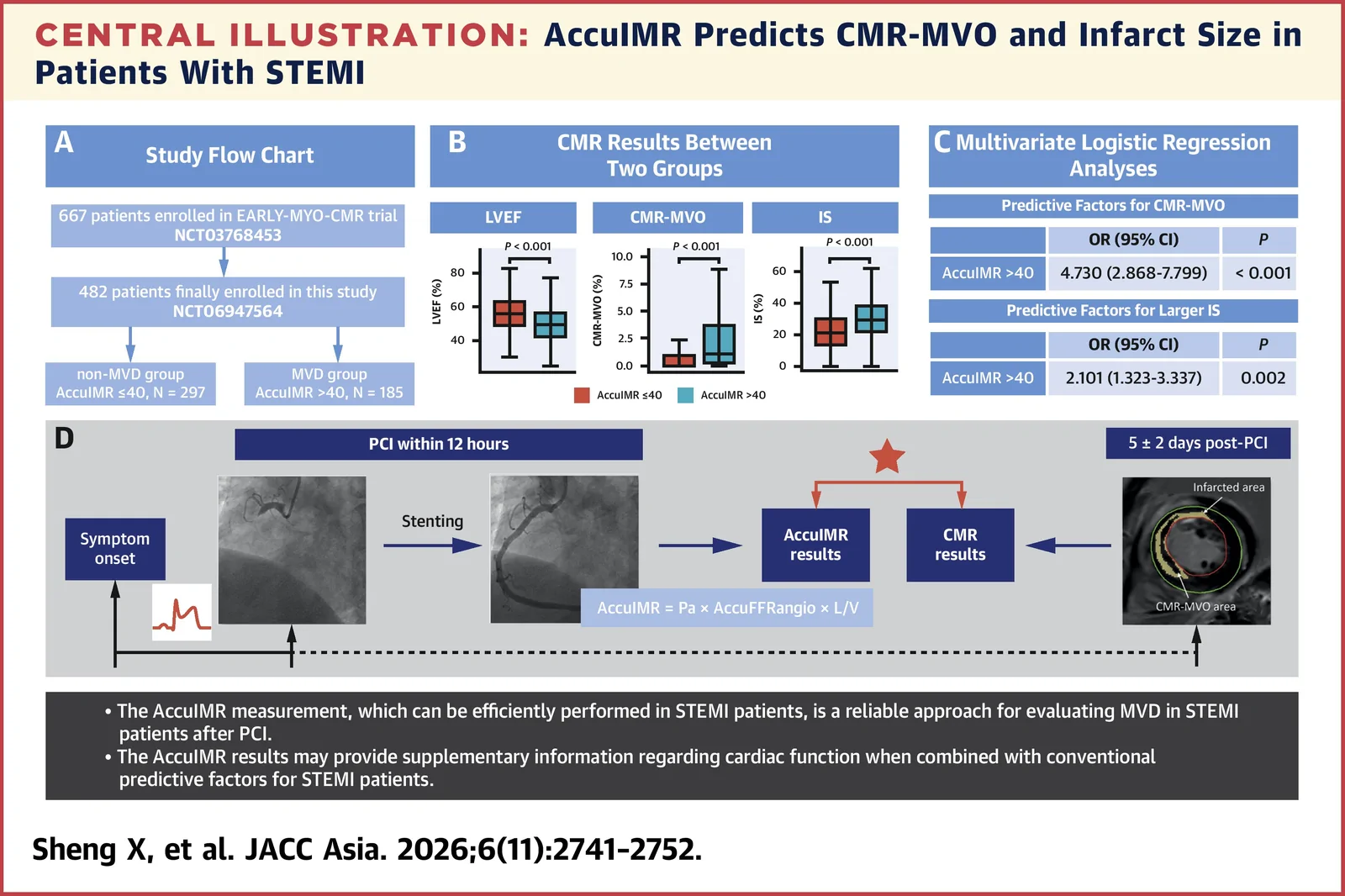
AccuIMR Predicts CMR-MVO and Infarct Size in Patients With STEMI (A) Study flow chart. (B) CMR results between the 2 groups. (C) Multivariate logistic regression model analyses. (D) Study protocol diagram. AccuIMR = angiography-derived index of microcirculatory resistance; CMR = cardiac magnetic resonance; CMR-MVO = cardiac magnetic resonance–derived microvascular obstruction; IS = infarct size; LVEF = left ventricular ejection fraction; MVD = microvascular dysfunction; PCI = percutaneous coronary intervention; STEMI = ST-segment elevation myocardial infarction.

With the maturation of 3D reconstruction techniques for coronary vessels, several simulated IMR methods based on angiography have been put forward. Three mainstream simulated IMR methods, supported by clinical evidence, were identified: angio-IMR (RainMed),^21^^,^^23^ AccuIMR (ArteryFlow Technology),^12^^,^^13^ and IMRangio (Medis).^24^^,^^25^ All of these methods share a common conceptual framework, yet they differ in their procedural approaches.

Among the 3 methods, AccuIMR employs 2 angiographic projections separated by at least 25°, enabling more accurate 3D reconstruction of the target vessel with deep learning algorithms. Unlike approaches based on a fixed vessel length, the calculation of AccuIMR incorporates the actual vessel length, making it more broadly applicable. In addition, instead of assuming the blood pressure, AccuIMR integrates the measured mean aortic pressure obtained during angiographic imaging. Theoretically, these features suggest that AccuIMR may align more closely with wire-based IMR. Consequently, AccuIMR has demonstrated excellent diagnostic performance across patients with chronic coronary syndrome, non–ST-segment elevation myocardial infarction (NSTEMI), and STEMI.^12^^,^^13^

However, because of the high cost of the IMR wire and the potential risks associated with the IMR test in STEMI patients, all accuracy studies using wire-based IMR as the reference standard consistently have a small sample size among STEMI patients. As a result, these accuracy studies are inadequate for establishing the clinical value of simulated IMR methods. Additional studies with a larger sample size are essential to further verify the clinical value of AccuIMR in STEMI patients.

CMR-MVO effectively reflects microvascular function in STEMI patients and is widely recognized as a powerful predictor of adverse clinical outcomes. Moreover, CMR is considered the gold standard for accurately quantifying IS after STEMI. Given these characteristics, CMR is appropriate to serve as a reference standard for simulated IMR methods.^26^^,^^27^ Accordingly, we adopted CMR results as the reference standard in our investigation. Specifically, CMR-MVO was used to represent MVD, whereas IS and LVEF were adopted to indicate cardiac function.

This study included a total of 522 STEMI patients with angiographic images and adequate CMR results. Among these 522 cases, 482 patients successfully underwent AccuIMR measurement based on the initial angiographic images. The relatively large sample size ensured the reliability of the study conclusion to a certain degree. Moreover, considering that this is a retrospective study and all angiographic images were previously collected without any specific selection, the success rate of AccuIMR measurement was notably high. This indicates that the AccuIMR measurement is relatively easy to perform.

The rate of MVD, classified according to the AccuIMR value (AccuIMR >40), was approximately 38.4%. In the MVD group, the CMR-MVO rate was 80.5%, while in the non-MVD group, it was 46.1%. These results are consistent with prior studies comparing wire-based IMR and CMR.^9^ This may to some extent indicate the consistency between wire-based IMR and AccuIMR.

The CMR performed postreperfusion revealed that patients in the MVD group had significantly larger areas of IS and CMR-MVO and lower LVEF compared to the non-MVD group. Multivariate logistic regression analysis identified the AccuIMR value as an independent predictive factor for both CMR-MVO and larger IS. Collectively, these findings indicate that patients with higher AccuIMR values are likely to have a greater prevalence of MVD and poorer cardiac function. Other research regarding the long-term prognosis using AccuIMR in diverse populations has also yielded similar results.^28^, ^29^, ^30^

LAD as the culprit vessel was an independent predictive factor for a larger IS, yet was not an independent predictive factor for CMR-MVO. This finding aligns with previous research.^16^^,^^31^^,^^32^^,^^33^

Collectively, DeLong's test, NRI/IDI, and likelihood ratio test indicated that AccuIMR may offer supplementary clinical information when combined with conventional predictive factors for STEMI patients. The practical implementation of this noninvasive test to assist clinicians in treating STEMI patients demands further exploration through clinical practice.

### Study limitations

Firstly, this study retained a retrospective and observational character. Prospective investigations may potentially offer further validation of AccuIMR. Secondly, we only analyzed CMR data within 7 days after reperfusion. Long-term data, which might contain crucial prognostic information, were not incorporated into this study. Other research using the MACE (Major Adverse Cardiovascular Events) dataset could supplement the findings of this study. Third, wire-based IMR was not performed in this study, and the accuracy of AccuIMR was only verified through the overall and grouped proportions. Although prior research had already established a strong correlation between AccuIMR and wire-based IMR, the absence of wire-based IMR in this study may still introduce errors.

## Conclusions

The AccuIMR measurement, which can be efficiently performed in STEMI patients, is a reliable approach for evaluating MVD in STEMI patients after PCI. The AccuIMR results may provide supplementary information regarding cardiac function when combined with conventional predictive factors for STEMI patients.

## Funding Support and Author Disclosures

This work was supported by the Shanghai Municipal Health Commission “Top Priority Research Center” (grant No. 2023ZZ02021); the Investigator-initiated Trial Program of Shanghai Pudong New Area Health Commission (the Cohort Study Program; 2025-PWDL-07); the Medical Discipline Construction Program of Shanghai Pudong New Area Health Commission (the Key Disciplines Program; PWZxk2025-07); and the Punan Branch of Renji Hospital (PN2023A1). These sponsors provided support for this study during the data collection and analyses. The authors have reported that they have no relationships relevant to the contents of this paper to disclose.
